# Community Structure and Biodiversity of Active Microbes in the Deep South China Sea

**DOI:** 10.3390/microorganisms12112325

**Published:** 2024-11-15

**Authors:** Taoran Yang, Yinghui He, Ming Yang, Zhaoming Gao, Jin Zhou, Yong Wang

**Affiliations:** 1 Institute for Ocean Engineering, Shenzhen International Graduate School, Tsinghua University, Shenzhen 518055, China; 2 Shenzhen Key Laboratory of Advanced Technology for Marine Ecology, Shenzhen International Graduate School, Tsinghua University, Shenzhen 518055, China; 3 Institute of Deep Sea Science and Engineering, Chinese Academy of Sciences, Sanya 572000, China

**Keywords:** MISNAC, in situ, deep sea, transcriptome

## Abstract

The deep ocean harbors a group of highly diversified microbes, while our understanding of the active microbes that are real contributors to the nutrient cycle remains limited. In this study, we report eukaryotic and prokaryotic communities in ~590 m and 1130 m depths using 16S and 18S rRNA Illumina reads (miTags) extracted from 15 metagenomes (MG) and 14 metatranscriptomes (MT). The metagenomic 16S miTags revealed the dominance of Gammaproteobacteria, Alphaproteobacteria, and Nitrososphaeria, while the metatranscriptomic 16S miTags were highly occupied by Gammaproteobacteria, Acidimicrobiia, and SAR324. The consistency of the active taxa between the two depths suggests the homogeneity of the functional microbial groups across the two depths. The eukaryotic microbial communities revealed by the 18S miTags of the metagenomic data are dominated by Polycystinea; however, they were almost all absent in the 18S metatranscriptomic miTags. The active eukaryotes were represented by the Arthropoda class (at 590 m depth), Dinophyceae, and Ciliophora classes. Consistent eukaryotic communities were also exhibited by the 18S miTags of the metatranscriptomic data of the two depths. In terms of biodiversity, the ACE and Shannon indices of the 590 m depth calculated using the 18S metatranscriptomic miTags were much higher than those of the 1130 m depth, while a reverse trend was shown for the indices based on the metagenomic data. Our study reports the active microbiomes functioning in the nutrient utilization and carbon cycle in the deep-sea zone, casting light on the quantification of the ecological processes occurring in the deep ocean.

## 1. Introduction

The deep ocean serves as a vast habitat for microbiomes. Microbial cell density in the water column decreases with water depth, but the deep ocean, nonetheless, contains about 55% of the total microbial cells in the pelagic ocean [1]. There is an increasing understanding of the phylogenetic diversity and functional diversity of microbiomes in the deep ocean [2,3]. The mesopelagic zone of the ocean is located between a depth range of 200–1000 m. It is a transition layer of carbon vertical cycling and serves as a huge reservoir of marine life with a high diversity [4,5,6]. The Tara Ocean project has revealed global mesopelagic and bathypelagic biodiversity and microbial communities using 16S rRNA gene amplicons, metagenomics, and metatranscriptomics [7]. However, efforts to investigate microbial activity in the deep sea are rare, especially for the active microbial groups with real contributions to sustain the deep-sea ecosystem. The previous studies revealed the dominance of Proteobacteria and Polycystinea in the deep-sea water column [8]. Undoubtedly, some of the taxa were dying or staying a low metabolic activity with the DNA-based methods [9,10,11]. The gene expression level of these dominant taxa remains a question at present.

To ensure an accurate evaluation of the ecological contribution of the deep-sea microbiomes, we need high-quality metatranscriptomes for deep-sea samples. However, due to a technical restriction in large-volume sampling and low biomass from the deep sea, a low amount of degrading RNA was obtained during the sample recovery. Biases in metatranscriptomes are also likely to occur during collection and preservation, particularly in microbial communities from deep-sea habitats, where in situ conditions can significantly differ from surface [12]. Therefore, detecting the in situ activities of the deep-sea microorganisms is still challenging regardless of the alteration of gene expression profile during sample treatment onboard [13]. The large-volume in situ filtration of deep-sea water requires an automatic device to ensure less disturbance on microbial community and the metabolic activity of the deep-sea inhabitants [14]. In the hadal zone (>6000 m depth), in situ filtered water was used for metagenomics and metatranscriptomics work, which indicated the metabolic activities of a distinct microbial spectrum involved in dissolved organic matter degradation [15]. Similarly, AFIS was applied to detect much higher ammonia oxidation efficiency using in situ fixed microbial cells from the deep ocean [12,16]. With the invention and application of a Multiple in situ Nucleic Acid Collection (MISNAC) device for the sampling of nucleic acids directly, we are able to obtain microbial RNA within different time intervals for the investigation of active microbes [17]. For instance, the metabolic profiles of different SAR202 lineages have been detected with the metatranscriptomes for the samples obtained by the MISNAC subunits [18], and a high diversity of Ciliophora, Discoba, and Copepoda protists has been revealed with in situ metagenomes [19]. However, what are the community structures and biodiversity of active microbiomes in the mesopelagic and bathypelagic zones have not been answered. They probably play similar ecological roles if the active taxa were consistent across depths and stations.

Usually, microbial community composition was revealed with 16S rRNA gene amplicons (DNA), while the potential transcriptional activity of the microbes was estimated by analyzing the relative abundance of 16S rRNA gene amplicons (RNA). Nowadays, high-throughput sequencing-based metagenomes (DNA-based) are used to determine microbial community composition and functional potential. Both metagenome-assembled genomes and 16S Illumina sequencing reads (miTags) are used for the detection of microbial communities [20]. Furthermore, the miTags of metatranscriptomes (RNA-based) provide a proxy for assessing the community structures of active microbes, and surveying its activity is important to understanding the real ecological players [21,22,23]. The communities estimated using the relative abundance of 16S rDNA and rRNA reads in metagenomes and metatranscriptomes, respectively, differ a lot within an environmental sample. This method can be applied to distinguish the active and inactive microbes [24,25,26]; however, it has not been used to investigate microbial communities in the deep ocean on a large scale. More importantly, the samples for previous studies were not collected in situ [8,24,27], which might result in a disturbed transcriptome of the active microbes.

The present study addresses this gap by utilizing the MISNAC device to collect in situ samples of metagenomic and metatranscriptomic data of the mesopelagic and bathypelagic microorganisms in the northwestern South China Sea (SCS). From the metagenomes and metatranscriptomes for about 590 m and 1130 m depths, we obtained high-throughput 16S and 18S miTags, followed by the bioinformatics and statistical analysis of the deep-water microbiota. We elucidated the microbial diversity and community structures of total microbes (DNA-based communities, hereafter ‘abundant’ communities) and microbes with potential for protein synthesis (RNA-based communities, hereafter ‘active’ communities). Consistent active microbes were revealed by the metatranscriptomic data at two mesopelagic depths and a depth close to the upper boundary of the bathypelagic layer, regardless of the different communities shown in the metagenomic data. The higher biodiversity of active microbes at ~590 m depth samples, compared with that at 1130 m depth, indicates a microbial relationship that much differs from the previous works and within different deep-sea layers. Our study, therefore, shows a functional microbial assembly in the deep-sea zone of SCS that is vital for the vertical transmission of nutrients and carbon.

## 2. Materials and Methods

### 2.1. In Situ Sampling and Environmental Factors

**Figure 1:**
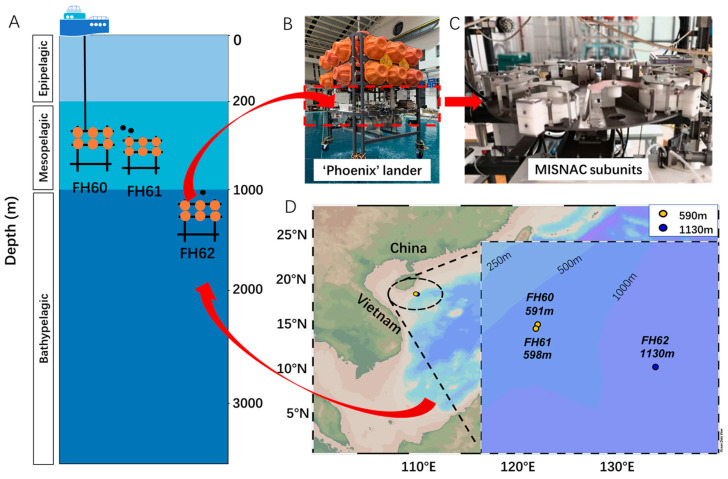
Sampling sites and method. (**A**) Three deep-sea lander deployments with the MISNAC apparatus obtained a total of 15 samples at depths of approximately 590 m and 1130 m. (**B**) A deep-sea lander named ‘Phoenix’ equipped with (**C**) sampling device MISNAC. (**D**) The sampling sites of the cruise in the South China Sea.

In situ deep-sea samples were collected at the depths of 591 m, 598 m, and 1130 m using MISNAC subunits during three deep-sea lander deployments (FH60, FH61, and FH62) in the same region in March of 2022 (Figure 1, Table S1). The MISNAC subunits were used for the time-series sampling, and each subunit filtered approximately 40–80 L of water in about 4 h, capturing microorganisms within the size range of 0.22 µm to 1 mm, followed by in situ cell lysis in the filtration chambers (Figure 1C). The membranes in the filtration chambers were immediately stored at −20 °C in a refrigerator after the recovery of the lander.

The CO_2_ concentration was measured using a CO_2_ sensor (Pro-Oceanus, Bridgewater, NS, Canada), which was also mounted on the deep-sea lander. The total organic carbon (TOC), total organic nitrogen (TON), NO_2_^−^, NH_4_^+^, and NO_3_^−^ were measured using the deep-sea water samples collected by Niskin bottles simultaneously equipped on the deep-sea lander.

All the sample IDs were prefixed with ‘FH’ (derived from Feng Huang (The Phoenix), the name of the lander). FH60, FH61, and FH62 refer to the three lander deployments at the depths of 591 m, 598 m, and 1130 m, respectively (Figure 1A,B). Although the MISNAC apparatus could complete the nucleic acid collection of nine water filtrations in one launch, only up to 6 of the MISNAC units were used in this study in one launch due to time limitations in the cruise (Figure 1C). The numbering connected by hyphens corresponds to the chronological order of the samples collected by the different MISNAC subunits.

### 2.2. Nucleic Acid Extraction and Sequencing

Total DNA and RNA were extracted from the 15 MISNAC filtration membranes using a DNA/RNA co-extraction kit (Tiangen, Beijing, China) according to the manufacturer’s instructions [28]. DNA concentrations were evaluated using a Qubit 4.0 Fluorometer (Thermo Fisher Scientific, Waltham, MA, USA). For DNA library preparation, metagenomic high-throughput libraries were constructed using the VAHTS Universal DNA Library Prep Kit for Illumina V3 (Vazyme, Nanjing, China). Sequencing was performed on an Illumina NovaSeq 6000 platform (Illumina, San Diego, CA, USA) with a 2 × 150 bp paired-end run.

For the total RNA, double-stranded cDNA was synthesized following the instructions of the Ovation RNA SEQ System V2 Kit (Qiagen, Hilden, Germany). To construct a high-throughput metatranscriptome library for Illumina, the VAHTS Universal V3 DNA Library Prep Kit (Vazyme, Nanjing, China) was employed. The libraries were sequenced on an Illumina NovaSeq 6000 platform (Illumina, San Diego, CA, USA) with a 2 × 150 bp paired-end run.

### 2.3. Quality Control, OTU Clustering, and Taxonomic Profiling

Raw reads were trimmed to remove adapters and then filtered using fastp (v.0.23.1) [29]. A quality control was performed using FastUniq (v.1.1) [30] with default parameters. Reads containing adaptors, low-quality reads (assigned by a quality score <20 for >30% of the read length), or unpaired high-quality reads were removed. After the quality control, one of the metatranscriptomic samples collected at a depth of 591 m (FH60-3-MT) was excluded from the subsequent analyses because of exceptionally low sequencing depth. The ribosomal RNA (rRNA) Illumina sequencing reads (miTags) were identified from the clean metagenomic and metatranscriptomic reads using rna_hmm3.py, which employed HMMER (v.3.1b2) [31] to identify ribosomal RNA gene fragments from both forward and reverse metagenomic reads. 16S miTags (≥100 bp) were recruited by an in-house Python script [32] and were matched to the variable region V4 of the 16S rRNA by HMMsearch (https://github.com/AlenaYoung/2022SCS-microbes/tree/master, 9 November 2024) [33]. Similarly, 18S miTags were also extracted from clean metagenomic and metatranscriptomic reads and were matched to the V9 region of the 18S rRNA gene.

16S and 18S miTags were processed following the QIIME2 pipeline [34]. They were then clustered into operational taxonomic units (OTUs) with ≥97% similarity using the VSEARCH pipeline, separately [35]. The tag sequence with the highest abundance was selected as the representative sequence for each OTU. The taxonomic assignment of the 16S miTag OTUs was conducted using the Feature-classifier classify-sklearn command in QIIME2 against the Silva (release 138) database [36], while the 18S miTag OTUs were assigned to eukaryotes against the PR2 (v.5.0.0) database [37].

### 2.4. Statistic Analysis

All the following statistical analyses and visualizations were performed with the R software (v.4.3.2) [38]. The outputs from QIIME2, including the table of OTUs, taxonomy, and tree files were processed by the R package phyloseq [39]. To normalize the sequencing depth for different samples, the OTU table was transformed to relative abundance using the phyloseq function transform_sample_counts. Metagenomic and metatranscriptomic miTags (hereafter refering to 16S and 18S miTags extracted from metagenomes and metatranscriptomes, respectively) were selected from the total table for subsequent analysis.

A Venn diagram was used to visualize the distribution of OTUs across the 15 metagenomic and 14 metatranscriptomic seawater samples using the ‘ggvenn’ package in R. The accumulation curve of the detected OTUs in the metagenomes and metatranscriptomes was calculated using the ‘vegan’ package [40] in R with 100 permutations.

ACE and Shannon indices were used to evaluate the alpha diversity of the microbial communities using the ‘vegan’ package in R. Bray–Curtis dissimilarity matrix and principal coordinate analysis (PCoA) were used to estimate and visualize the beta diversity of the samples using the ‘ape’ package in R [41]. The statistical analysis of the beta diversity was performed by the permutational multivariate analysis of variance (PERMANOVA). Community clustering for each sample was analyzed by the Bray–Curtis dissimilarity matrix using an unweighted pair-group method with arithmetic means (UPGMA).

## 3. Results

### 3.1. Community Structures of Active Deep-Sea Microbes from SCS Deep Layers

**Figure 2:**
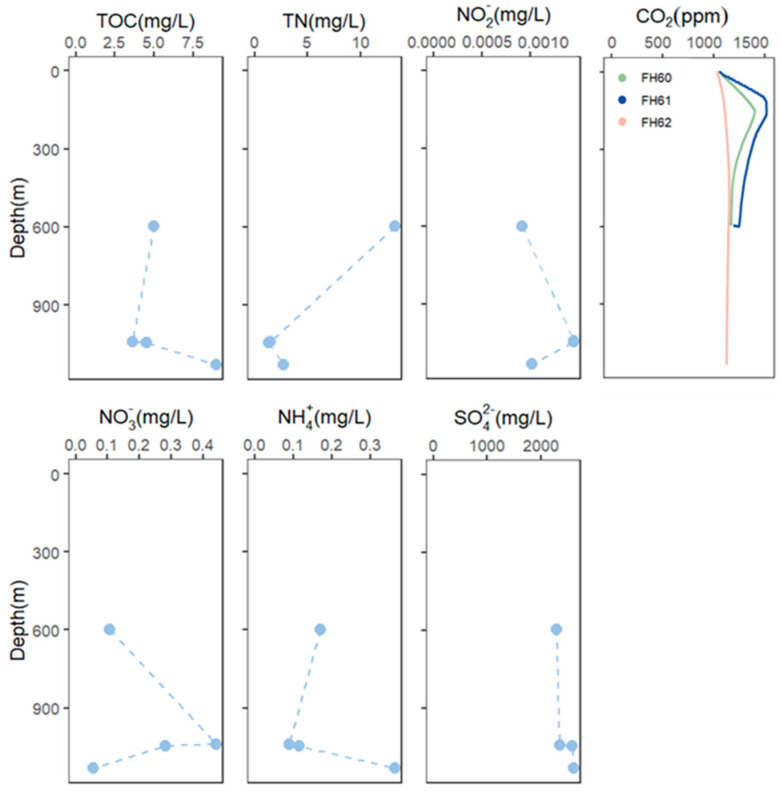
The physicochemical analyses were measured for the plots.

The water samples were in situ filtered using the MISNAC working units at two depths (~590 m for FH60 and FH61 and ~1130 m for FH62), collecting 15 samples at different time intervals for the subsequent metagenomics and metatranscriptomics work (Figure 1). In the FH60 and FH61 deployments, the peak of CO_2_ measurements at about 1400 ppm was observed at approximately 150 m depth. In the FH62, the CO_2_ measurements gradually increased to 1150 ppm (Figure 2). The TOC, TN, ammonia, nitrite, nitrate, and sulfate concentrations were also measured (Figure 2, Table S2).

**Figure 3:**
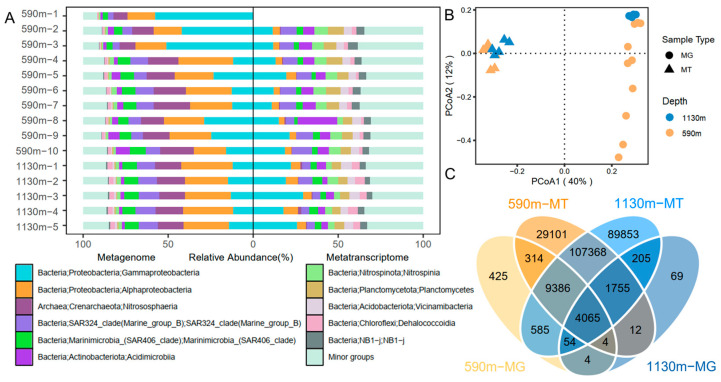
Relative abundance and diversity indices of the abundant and active prokaryotes based on the 16S miTags extracted from metagenomes (MG) and metatranscriptomes (MT). (**A**) The structures of the prokaryotic communities at the class level in the samples gathered at different depths. Minor groups contain all the small taxa with relative abundance <5%. (**B**) Venn plot showing the shared prokaryotic OTUs among the different depths. (**C**) Principal coordinates analysis (PCoA) plot using the prokaryotic community structures.

**Figure 4:**
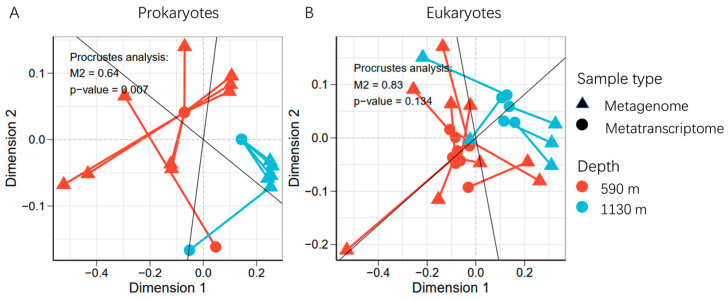
Procrustes analysis connecting the metagenome communities and metatranscriptome communities in the 590 m and 1130 m samples of (**A**) prokaryotes and (**B**) eukaryotes.

The 16S and 18S miTags were extracted from the sequencing data for the microbial communities and biodiversity analyses. A total of 15,609,878 16S miTags and 2,226,731 18S miTags sequences were identified, respectively, and these were clustered into 213,360 and 27,337 OTUs for prokaryotes and eukaryotes, respectively. The sequencing depth of the metagenomes and metatranscriptomes differed between the samples. Therefore, to minimize the impact of the sequencing depth, the metagenomic and metatranscriptomic 16S miTags were normalized to 8851 reads and 176,092 reads, while the metagenomic and metatranscriptomic 18S miTags were rarefied to 2423 reads and 12,243 reads, respectively (the lowest sequence number across all the samples) (Table S3). The rarefaction curves of the OTUs for the 16S miTags in both the metagenomes and metatranscriptomes showed a plateau (Figure S1), indicating a sufficient sequencing depth for the identification of all the species in the sampling stations. For the prokaryotic communities (Figure 3A), Gammaproteobacteria, Alphaproteobacteria, SAR324, Nitrosophaeria, and Marinimicrobia dominated the communities based on the metagenomic 16S miTags. From the ~590 m to the ~1130 m depth, the relative abundance of Gammaproteobacteria decreased significantly (*t*-test; *p* < 0.05). Using the 16S miTags in the metatranscriptomes, the most active prokaryotes were inferred to be Gammaproteobacteria, Acidimicrobiia, Alphaproteobacteria, SAR324, Marinimicrobia, Planctomycetes, and Vicinamibacteria. There were significantly more active minor groups detected by the metatranscriptomic 16S miTags (*t*-test; *p* < 0.05). Compared with the community structures based on the metagenomic 16S miTags, those based on the metatranscriptomic 16S miTags were highly consistent regardless of the sampling depth difference, as reflected by a PCoA plotting using the community structures (Adonis, *p* = 0.001, numbers of permutations = 1000) (Figure 3B). In contrast, the communities of the ~590 m depth differed much as revealed by the classification of their metagenomic 16S miTags. The OTUs shared among the metagenomic and metatranscriptomic datasets of the two depths were counted and shown in a Venn diagram (Figure 3C). The number of the shared OTUs showed that (1) 9.06% of the ~590 m metagenomic OTUs were present in the ~590 m transcriptomic OTUs; (2) 2.85% of the 1130 m metagenomic OTUs were present in the ~1130 m transcriptomic OTUs; (3) the percentage (48.12%) of OTUs shared among the metatranscriptomic OTUs were much higher than that (17.82%) shared among the metagenomic OTUs. A Procrustes analysis based on the Bray–Curtis distances revealed a difference and significant correlation between the metagenome and metatranscriptome community composition (M2 = 0.64, *p* = 0.007) (Figure 4A). This result is in agreement with that shown by the PCoA plot.

### 3.2. Diversity and Composition of Active Eukaryotic Microbes in the Deep Water

**Figure 5:**
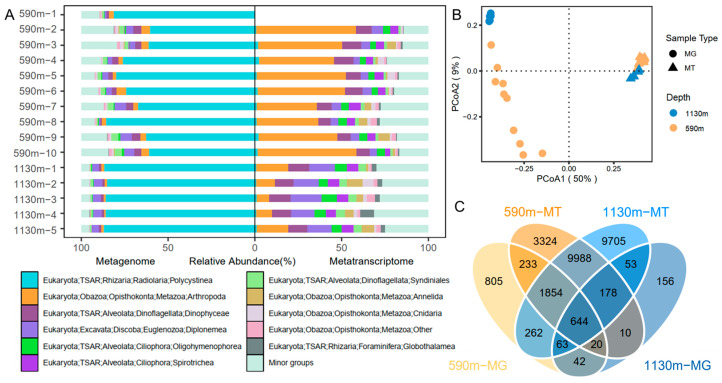
Relative abundance and diversity indices of the abundant and active eukaryotes based on the 18S miTags extracted from metagenomes (MG) and metatranscriptomes (MT). (**A**) The structures of the eukaryotic communities at the class level in the samples gathered at different depths. Minor groups contain all the small taxa with relative abundance <5%. (**B**) Venn plot showing the shared eukaryotic OTUs among the different depths. (**C**) Principal coordinates analysis (PCoA) plot using the eukaryotic community structures.

In light of the taxonomic classification of the eukaryotic OTUs, the most abundant eukaryotic class was Polycystinea (Rizaria and Radielaria) in the metagenomic 18S OTUs, particularly in the ~590 m depth (Figure 5A). However, they were almost all inactive as revealed by the metatranscriptomic 18S OTUs. An obvious trend is a significant difference between the relative abundances of Polycystinea in the metagenomic OTUs and those in the metatranscriptomic ones (Figure 5A), indicating that there are more inactive Polycystinea in the deep ocean. Except for the Polycystinea, all the other eukaryotes were abundantly present in the metatranscriptomic communities, including Arthropoda (especially Maxillopoda X and Ostracoda at genus level), Dinophyceae (Dinoflagellata), Diplonemea (Euglenozoa), Oligohymenophorea, and Spirotrichea (Ciliophora). Similarly, the eukaryotic community structures based on the metatranscriptomic OTUs resembled each other, in strong contrast to the divergence between the structures based on the metagenomic OTUs (Figure 4B and Figure 5B). In a Venn diagram, at the OTU level, 16.93% of the eukaryotic OTUs were shared for the miTags extracted the ~590 m and 1130 m metatranscriptomes (Figure 5C), which is much higher than the percentage (n = 4.12%) using the two sets of metagenomic 18S miTags.

### 3.3. Alpha Diversity Analysis Provided a Contrast Pattern Between the Metagenomic and Metatranscriptomic OTUs

**Figure 6:**
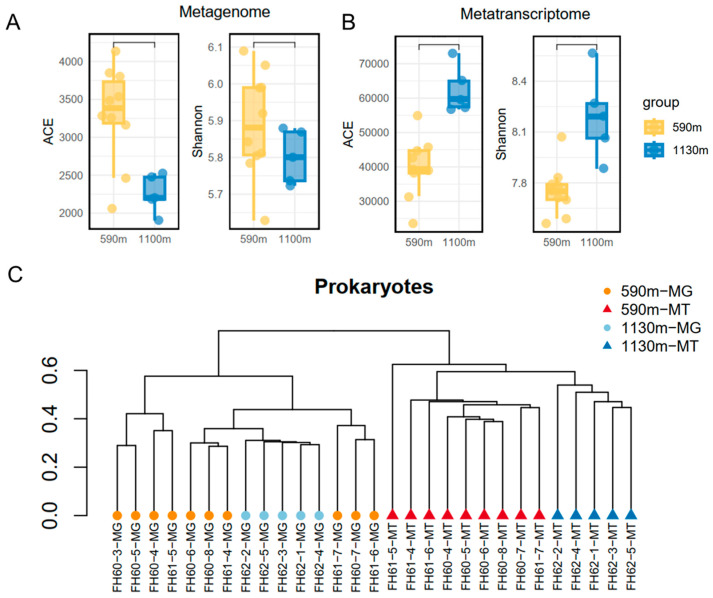
Alpha diversity and UPGMA tree of the abundant and active prokaryotes based on the 16S miTags. (**A**) Alpha diversity indices for the prokaryotic communities extracted from metagenomes (MG). (**B**) Alpha diversity indices for the prokaryotic communities extracted from metatranscriptomes (MT). (**C**) UPGMA tree generated from the Bray–Curtis dissimilarity matrix of the abundant and active prokaryotes for the different depths. The IDs with ‘MG’ refer to the communities derived from the 16S metagenomic miTags; those with ‘MT’ denote those derived from the 16S metatranscriptomic miTags.

Alpha diversity was analyzed using the miTags to differentiate between the metagenomic and metatranscriptomic datasets. Rarefaction curves indicate that there are slightly more OTUs in the ~590 m metagenomic miTags compared with those of the ~1130 m counterparts (Figure S1). A reverse trend was observed for the transcriptomic datasets. At least ten-fold more OTUs were revealed in the metatranscriptomic miTags than those of the metatranscriptomic miTags. Alpha diversity indices, ACE and Shannon, showed a similar result (Figure 6A,B). Compared to the datasets for the ~1130 m depth, higher ACE and Shannon indices were detected in the metagenomic datasets of the ~590 m depth, while lower indices were shown in the metatranscriptomic dataset at this depth. A UPGMA clustering analysis based on the Bray–Curtis dissimilarity matrix showed a higher similarity between the ~590 m and 1130 m metagenomic miTag-based communities compared with those based on the metatranscriptomic miTags (Figure 6B). A mixture of the different communities based on the 16S metagenomic miTags from the different sampling depths was discerned, while the clusters consisting of their metatranscriptomic miTag-based prokaryotic communities for the ~590 m and ~1130 m depths were clearly separated.

**Figure 7:**
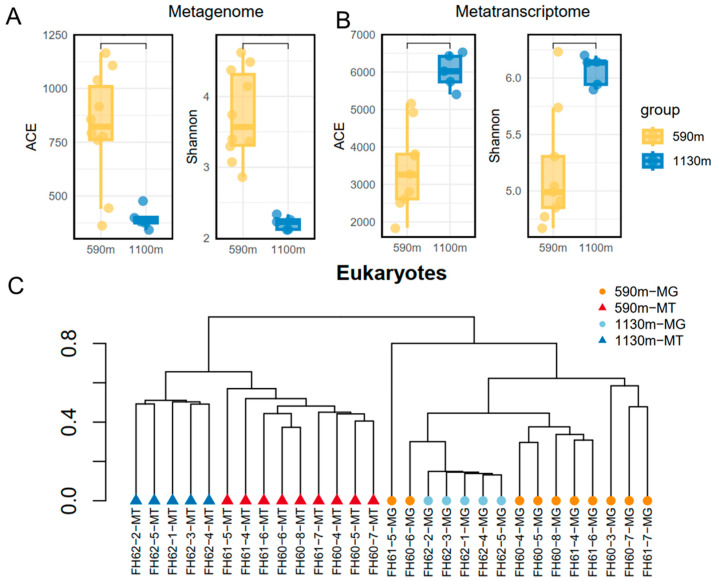
Alpha diversity and UPGMA tree of the abundant and active eukaryotes based on the 18S miTags. (**A**) Alpha diversity indices for the eukaryotic communities extracted from metagenomes (MG). (**B**) Alpha diversity indices for the eukaryotic communities extracted from metatranscriptomes (MT). (**C**) UPGMA tree generated from the Bray–Curtis dissimilarity matrix of the abundant and active eukaryotes for the different depths. The IDs with ‘MG’ refer to the communities derived from the metagenomic 16S miTags; those with ‘MT’ denote those derived from the 16S metatranscriptomic miTags.

For the alpha diversity indices of the eukaryotic communities, the ACE and Shannon of the 590 m communities were almost four-fold higher than those of the 1130 m communities (Figure 7A,B). At the metatranscriptomic level, about two-fold higher ACE and Shannon indices were revealed for the metatranscriptomic miTag-based communities at the 1130 m depth compared with the indices for the communities at the ~590 m depth. A UPGMA tree built by using the Bray–Curtis dissimilarity matrix of the eukaryotic communities indicates a high divergence of the communities between the metatranscriptomic datasets for the sampling depths and a mixture of the communities in the tree for the metagenomic datasets of the different depths (Figure 7C).

## 4. Discussion

In the deep ocean, the microbial communities revealed by the 16S rRNA gene amplicons and metagenomics differ from those of the active microbes [42]. Here, we explored RNA and DNA samples as a comprehensive way to discover the active prokaryotic and eukaryotic microbiomes in the mesopelagic and bathypelagic zones of the northwestern SCS. The microbial communities were much more dynamic in the metagenome-based communities [43], which indicates different vertical transmission rates of microbes and nutrient inputs from the surface. For the sampling station on a continental slope, the upward migration of the microbes following the water flow might affect the dominant species of the water column [44]. For our study, the highly consistent active microbes in the metatranscriptome-based communities for the two zones suggest a homogeneity in the structures of microbial communities with metabolic activities as reflected by their transcriptional profiles of the 16S and 18S rRNA genes. Yet, there are limitations of this approach in accurately delineating active prokaryotes, as factors such as different life strategies can influence gene expression [9,45]. However, despite these limitations, the simultaneous characterization of 16S rRNA transcripts and genes provides an opportunity to identify the most active members of the community, as RNA serves as an indicator of the recent or current potential for protein synthesis. Therefore, our study showed the active microbes of different depths in the spring of the northwestern SCS.

In this study, we revealed the prevalence of Polycystinea in the samples for metagenomic studies. However, their rRNA gene transcripts were almost absent in the metatranscriptomic miTags, indicating their lack of activities in the deep-sea zone. With the number of DNA gene copies per collodarian cell being among the highest in eukaryotic lineages, along with potential cell breakage during collection, these rDNA copy numbers have likely resulted in an over-representation [46]. Similar results have been shown in our recent study using MISNAC as well [19]. Between the two layers of this study, active eukaryotic microbes slightly differed, particularly for Arthropoda represented by Maxillopoda. It has been reported that Maxillopoda is a major predator in the mesopelagic zone [47]. Their distribution seems limited by water depth, as we found at least two-fold more Maxillopoda at the 590 m depth compared to the 1130 m depth site. This is probably determined by the swimming ability of the Maxillopoda during diel vertical migration [48]. Ciliophora were a group of protists active in the deep sea [49]. Their richness and high activities were only shown by the in situ sampler MISNAC with a capacity of in situ nucleic acid extraction [19]. The sampling with the Niskin bottle probably caused cell explosion during the recovery of the bottle, probably due to the high amounts of dissolved gasses in the cells and the significant pressure difference from the deep sea to the surface [19].

There are many more active microbes in the deep sea, as we discovered that approximately 40% of the 16S miTags in the metatranscriptomes, which were assigned to the minor groups, were less represented in their genomic DNA in the metagenomes. This is likely causal to the exclusion of the abundant 16S rRNA gene reads for the inactive prokaryotic species in the metagenomic reads from the metatranscriptomic sequencing reads. Therefore, the really active microbes that are the dominant contributors in the deep-sea ecosystem were unfortunately ignored by the previous work. The ecological network of the deep ocean, therefore, is much more complicated than our previous estimate. This emphasizes the application of in situ sampling methods for the detection of the real ecological players.

Time-series samples were obtained by the MISNAC subunits for the deep-sea samples of different time intervals in this study. However, the diel rhythm was only reported in the surface ocean [26,50]. In the deep sea, such an obvious rhythm has not been reported yet. In the 1130 m depth of this study, we could not visualize a regular pattern of microbial changes across different time intervals. The low Polycystinea relative abundance in our metagenomic data was shown in different time intervals of the two sampling days at similar depths. At the ~1130 m depth, the community structures of the prokaryotes and eukaryotes resembled each other, indicating that the microbial community was stabilized under relatively constant environmental factors in this deep layer. With more MISNAC samples from different time intervals, depths, and stations globally, future studies will be able to provide more mechanistic insights into the potential regular pattern of active microbes in terms of the community structure and gene transcription of the deep ocean.

## 5. Conclusions

The active deep-sea microbes are still mysterious as it is still challenging to obtain high-quality well-preserved samples for metatranscriptomics work. In this study, we relied on the MISNAC samples to show the microbial community and biodiversity of the active microbes in the mesopelagic and bathypelagic zones at up to ~1130 m depth. Our results showed a high diversity of active microbes that differed from those identified by metagenomics methods. Therefore, our study paves the way to the discovery of the critical deep-sea indigenous inhabitants attributable to the formation of the distinct ecosystems in various depths and geological settings of the ocean. Our future efforts will provide the gene expressional profiles of the functional genes to reveal the metabolic network and the contribution of the key players in deep-sea ecosystems.

## Data Availability

The miTags are being deposited to NODE and can be accessed before the manuscript is officially published.

## Supplementary Materials

The following supporting information can be downloaded at https://www.mdpi.com/article/10.3390/microorganisms12112325/s1, Figure S1: Rarefaction curves for metagenomic (rDNA) and metatranscriptomic (rRNA) data; Table S1: Sampling stations and time interval; Table S2: Nutrient contents collected by Niskin bottle; Table S3: The number of rarefied reads; OTUs at the 97% similarity level.
