# Supplementary material for: Community Structure and Biodiversity of Active Microbes in the Deep South China Sea
Source: Microorganisms. 2024 Nov 15;12(11):2325. doi: 10.3390/microorganisms12112325 (PMC11596837; doi:10.3390/microorganisms12112325)
Supplement: Supplementary file 1 [file microorganisms-12-02325-s001.zip › microorganisms-3268082-supplementary.pdf]

Supplementary Figure S1

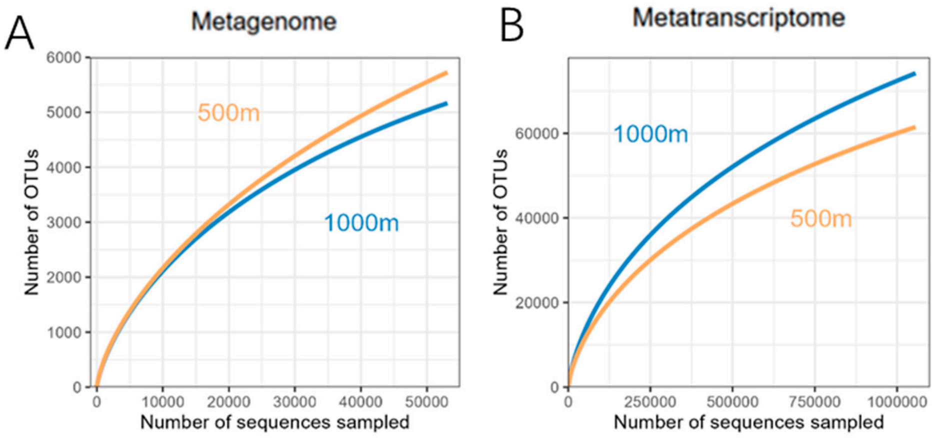

Supplementary Figure S1: Rarefaction curves for (A) metagenomic (rDNA) and (B) metatranscriptomic (rRNA) data. Curves showing the number of reads as a function of the number of OTUs identified.

Supplementary Table S1: Sampling stations and time interval.

Cruise	Metagenome Samples	Metatranscriptome Samples	Date	Time interval	Depth (m)	Longitude (E)	Latitude (N)
FH60	\	FH60-3-MT	Mar.21 2022	10:00-12:00	591	110°39.987‘	17°53.374’
FH60	FH60-4-MG	FH60-4-MT	Mar.21 2022	14:00-16:00	591	110°39.987‘	17°53.374’
FH60	FH60-5-MG	FH60-5-MT	Mar.21 2022	17:00-19:00	591	110°39.987‘	17°53.374’
FH60	FH60-6-MG	FH60-6-MT	Mar.21 2022	21:00-23:00	591	110°39.987‘	17°53.374’
FH60	FH60-7-MG	FH60-7-MT	Mar.22 2022	0:00-2:00	591	110°39.987‘	17°53.374’
FH60	FH60-8-MG	FH60-8-MT	Mar.22 2022	4:00-6:00	591	110°39.987‘	17°53.374’
FH61	FH61-4-MG	FH61-4-MT	Mar.22 2022	20:00-23:00	598	110°39.888‘	17°53.216’
FH61	FH61-5-MG	FH61-5-MT	Mar.22 2022-<br>Mar.23 2022	0:00-3:00	598	110°39.888‘	17°53.216’
FH61	FH61-6-MG	FH61-6-MT	Mar.23 2022	4:00-7:00	598	110°39.888‘	17°53.216’
FH61	FH61-7-MG	FH61-7-MT	Mar.23 2022	8:00-11:00	598	110°39.888‘	17°53.216’
FH62	FH62-1-MG	FH62-1-MT	Mar.23 2022	19:00-23:00	1130	110°48.345‘	17°50.580’
FH62	FH62-2-MG	FH62-2-MT	Mar.23 2022-<br>Mar.24 2022	23:00-3:00	1130	110°48.345‘	17°50.580’
FH62	FH62-3-MG	FH62-3-MT	Mar.24 2022	3:00-7:00	1130	110°48.345‘	17°50.580’
FH62	FH62-4-MG	FH62-4-MT	Mar.24 2022	7:00-11:00	1130	110°48.345‘	17°50.580’
FH62	FH62-5-MG	FH62-5-MT	Mar.24 2022	11:00-15:00	1130	110°48.345‘	17°50.580’

Supplementary Table S2: Nutrient contents collected by Niskin bottle.

Depth (m)	TN (mg/L)	NO^{2-} (mg/L)	NH^{4+} (mg/L)	NO^{3-} (mg/L)	TOC (mg/L)	SO_4^{2-} (mg/L)
591	\	\	\	\	\	\
598	13.2	0.0	0.2	0.1	5.0	2284.0
1130	2.8	0.0	0.4	0.1	9.0	2600.5
1041	1.5	0.0	0.1	0.4	3.6	2352.8
1045	1.3	0.0	0.1	0.3	4.5	2573.0

Supplementary Table S3: The number of rarefied reads, OTUs at the 97% similarity level

	16S DNA	16S RNA	18S DNA	18S RNA
Total Reads	15,609,878	15,609,878	2,226,731	2,226,731
Assigned OTUs	213,360	213,360	27,337	27,337
Normalized reads	8,851	176,092	2,423	12,243
